# Factors affecting family planning literacy among women of childbearing age in the rural Lake zone, Tanzania

**DOI:** 10.1186/s12889-022-13103-1

**Published:** 2022-04-04

**Authors:** Mohamed Kassim, Faraja Ndumbaro

**Affiliations:** Information Studies Programme, University of Dar es Salaam, P. O. Box 35092, Dar es Salaam, Tanzania

**Keywords:** Family planning, Lake zone, Literacy, Health literacy, Women of childbearing age, Rural, Tanzania

## Abstract

**Background:**

Low uptake of various recommended modern family planning methods is associated with inadequate family planning literacy among potential beneficiaries of the methods. As such, understanding factors affecting family planning literacy is key to addressing this problem. This study, therefore, explored factors affecting family planning literacy among women of childbearing age in the rural Lake Zone of Tanzania.

**Methods:**

The study utilized an exploratory descriptive qualitative research approach using focus group discussions to collect data. A total of eight focus group discussion sessions were held to solicit information from childbearing age women involved in the study. Thematic analysis was used to analyze the data collected from the study participants.

**Results:**

Several factors were found to negatively affect the family planning literacy of women of childbearing age in the communities under review. These factors were low levels of education, religious affiliation, and low family income. Other factors that were also found to negatively affect women’s family planning literacy include fertility preference, negative perceptions of family planning, preference of unproven family planning methods, limited access to reliable sources of family planning information, household responsibilities, and poor male partner support on family planning matters.

**Conclusion:**

This study has identified a multitude of factors affecting the family planning literacy of women of childbearing age. These factors can limit the women’s capacity to make informed decisions on the utilization of modern family planning methods. Thus, addressing these factors is pivotal in increasing the women’s overall uptake of various recommended family planning methods and enhancing their reproductive health outcomes.

## Background

Family planning is essential in helping women and their male partners to decide freely on whether to have children, how many to have, and when to do so [1, 2]. It improves both maternal and child health, reduces the prevalence of unwanted pregnancies and unsafe abortions, prevents sexually transmitted infections, and enhances economic well-being of families [3–5]. In fact, family planning also promotes women’s sense of autonomy and their ability to make health decisions [6]. As a result, the United Nations (UN) has prioritized it to increase and sustain the utilization of family planning because of its importance in the attainment of sustainable development goals. In particular, emphasis has been put on universal access to a full range of safe and reliable family planning methods to help couples realize their rights to freely and responsibly decide the number and spacing of their children [2, 7].

According to the World Health Organization (WHO), almost 60% of women of childbearing age use using family planning globally [8]. Even though evidence shows a global increase in the use of family planning, especially in Asia (62%) and Latin America (67%), sub-Saharan Africa, Tanzania inclusive, paints a different picture as there is average of less than 20% month use of family planning [9]. Furthermore, less than 30% of women of childbearing age in sub-Saharan Africa are use family planning [8] with more than 200 million women wishing to prevent unwanted pregnancies yet not using these services [10].

Tanzania has witnessed a notable increase in the utilization of modern family planning methods among women from 7% in 1991 to more than 30% in 2015 [11–13]. Despite such growth, more than 20% of various forms of family planning needs among women of childbearing age are unmet in the country [14]. There is also a high rate of contraceptive discontinuation among women [15]. Furthermore, there is a large geographical variation in access to and use of family planning methods in the country with some regions having considerably lower usage than the national average [16].

Persistently low uptake of family planning has been evident more in the Lake zone of Tanzania (15%) than in any other part of the country [11, 12, 17]. This zone also has a high (33%) of unmet need for family planning compared to the rest of the country as many women in need of such services fail to access them [11, 17]. Such women remain susceptible to various reproductive health and socioeconomic problems. Evidence shows that the low uptake of family planning has contributed to high fertility rates [18], which is as one of the risk factors for maternal morbidity and mortality [19]. Women and their families are likely to face financial burdens resulting from costs of health services, as a result [4, 18].

Low uptake of family planning services in most sub-Saharan countries, Tanzania included, is associated with inadequacy health literacy among women of childbearing age [6, 20–22]. Health literacy refers to the degree to which people have the capacity to obtain, process, and understand basic health information and services needed to make appropriate health decisions [23, 24]. This concept is one of the most significant social determinants of health [25–27] and is, thus, essential in changing people’s attitudes towards accepting and taking various recommended family planning methods.

Evidence in literature shows that people with inadequate health literacy tend to be less knowledgeable of their health conditions, less likely to use preventive health care services, and more likely to be hospitalized [28]. Such individuals can also be easily misguided by incorrect sources of health information [29]. In the context of family planning, inadequate family planning literacy is reported to contribute to poor acceptance, wrong use, and low uptake of various recommended family planning methods [20–22, 30].

Inadequate health literacy, as reported in previous studies [31–33], is more prevalent among people in rural areas than those in urban ones. This appears to explain the reported low uptake of recommended family planning methods by most people in rural areas [15, 18, 22, 34, 35]. Research shows that, unlike their urban counterparts, women residing in rural areas are likely to be affected by all types of unmet needs for family planning services [22, 35]. Women in these areas are likely to identify inadequate health literacy as the main barrier to their uptake of recommended family planning methods [22].

Exploring factors affecting the family planning literacy of women residing in rural areas is crucial in addressing the problem of low uptake of various family planning methods among women of childbearing age in these areas. This study, therefore, explored the perspectives of women of childbearing age residing in rural Lake Zone, in Tanzania, on factors affecting their family planning literacy.

## Methods

This study employed an exploratory descriptive qualitative research approach to explore factors affecting the family planning literacy of women of childbearing age in rural Lake Zone of Tanzania. This zone comprises six regions namely Mwanza, Geita, Shinyanga, Mara, Simiyu, and Kagera. The zone has a comparatively lower (15%) uptake of family planning than the rest of the country [11, 12, 17]. From the six regions of the zone, four (Shinyanga, Simiyu, Mara, and Kagera) were purposively selected for the study. Many women living in the rural areas of these regions face problems associated with access to healthcare [13], hence their selection. From each of these regions, one district (Shinyanga Rural in Shinyanga, Bariadi in Simiyu, Musoma rural in Mara, and Bukoba rural in Kagera) was purposively selected for the study. These districts are mainly rural, a characteristic of interest in this study. Subsequently, one ward (Iselamagazi in Shinyanga rural, Dutwa in Bariadi, Kemondo in Bukoba rural, and Mugango in Musoma rural) was selected from each of the four study districts. These wards serve as administrative headquarters of the districts under review and, thus, contain a heterogeneous population in terms of education levels, occupation, and religious affiliation.

The study targeted women of childbearing age residing in the selected wards. Purposive sampling was used to select respondents for inclusion in the study. Woman aged between 15 to 49 years, living in rural areas, and capable of expressing themselves concerning the topic in question were included in the study. Local community health workers (CHWs) from the respective villages helped to identify potential households from which individuals meeting the set inclusion criteria were picked. The CHWs also helped to identify potential study participants in the selected households and establish trust between the researchers and study participants.

Data from the study participants were mainly collected using focus group discussions (FGDs) based on a guide initially developed in English and translated into Kiswahili for easy comprehension among the participants. The guide consisted of open-ended questions on participants’ perspectives concerning factors affecting their family planning literacy. In all, 8 FGDs sessions involving a total of 72 participants were held in the selected wards of the four study districts. The number of participants in each group ranged from 8 to 10. In each ward, a stratified sampling technique was used to group studied women in three strata based on levels of education, differences in age, and distance to health facilities. These are essential variables in assessment of health information literacy. The strata were used at the sampling stage to ensure that each group accommodate heterogeneous nature of the sub population. Meaning saturation [36, 37] was a criterion for reaching the study’s sample size as more FGDs were needed to understand factors affecting women’s family planning literacy. Prior to the commencement of the FGDs sessions, researchers presented to the participants the motive of the study and core objectives of the sessions. Also, researchers together with participants set ground rules for discussions including effective participation, speak one at a time, and respect for other people’s opinions. All 8 FGD sessions were conducted by the researchers. Each session lasted between 60 and 90 min. To ease participants’ attendance, the discussions were held at places of their convenience.

Measures such as identification and formulation of the research problem, selection of study participants, and accuracy in data collection, analysis, and interpretation helped to ensure trustworthiness of the study findings. Audio recordings and field notes captured all the discussions during data collections. These recordings then underwent verbatim transcription in Kiswahili, the language of researchers with participants, before being translated into English and reviewed by the researchers. After the review, the transcripts were imported, coded, and analyzed thematically using NVivo software (QSR version 12). Themes generated from the narratives of the FGDs were then organized to group similar ones from the transcriptions into a cluster. The themes are socio-demographic factors, fertility preferences, negative perceptions, and misinformation on family planning, use of unproven methods of family planning, limited access to reliable sources of family planning information, household responsibilities, and men’s involvement in family planning.

### Ethical considerations

Ethical clearance certificate with reference number NIMR.HQ/R.8a/Vol. IX/3185 was obtained from the National Health Research Ethics Review Committee (NHRERC) of the Tanzania National Institute for Medical Research (NIMR). Permission to conduct the study was also sought from the local authorities in the four selected regions. Written informed consent was obtained from the participants who were able to read while verbal informed consent wa obtained from the participants who could not write. Informed consent for minors (those aged below 18 years), was obtained from their parents/guardians. In addition to this, the minors were fully informed about the study and made aware that they were free to decide to participate on not. The consent process was approved by the ethics review committee (NHRERC). All the study participants were assured that their participation would be kept anonymous throughout the study.

## Results

A total 72 women of childbearing age from rural areas of the four selected regions participated in the FGDs. The ensuing discussions revealed several factors that affected family planning literacy of the study participants. These factors have been grouped into seven themes as described in sections that follow:

### Socio-demographic factors

Three socio-demographic factors namely education, household economic status, and religious beliefs were found to affect the family planning literacy of women of childbearing age in this study.

#### Education

There were mixed feelings from study participants about how education affects their family planning literacy. On the one hand, many women that took part in the FGDs attributed their inadequate family planning literacy to low levels of education among them and their male partners. The women argued that their low levels of education made it is difficult for them to comprehend family planning information from diverse sources. As a result, many of them failed to utilize available family planning services. Some women mentioned facing difficulties following the calendar method to prevent unintended pregnancies:

*… sometimes we are told to use a calendar as one of the family planning methods. However, this method might be good for those with adequate levels of literacy as it might be easier for them to follow their calendars. The problem is that it is difficult, especially for us who have not gone to school, to apply such a method as most of us do not know how to follow such a calendar* (FGD 2, Musoma).

On the other hand, other women, particularly literate ones, who had at least primary education, argued that education enabled them to know various family planning methods. The women reported that reading enabled them to follow family planning messages from different sources including those in print format. As a result, these women were interested in finding more information on various family planning methods before deciding on whether to use any one of them or not:

*Many educated and more informed people are using family planning to control the number of children they want to have. Thus, you may find them interested in finding information on different forms of family planning* (FGD 2 – Shinyanga rural).

#### Household economic status

The family’s economic status emerged as one of the determinants of women’s family planning literacy. Firstly, discussion participants reported that economic hardships a family with many children faces made it difficult for them to take care of all the children. Such hardships compelled women to consider using family planning to reduce the number of unintended pregnancies and have manageable family sizes. Thus, gathering information on family planning from different sources becomes crucial:

*Our incomes very small, but we have numerous children to take care of. So, we thought that now is the right time to start family planning. That is why we have started gathering information on it* (FGD 1 – Bukoba).

Study participants also linked household economic status to their families’ ability to own radio and television for getting family planning-related information. These women reported that their poor economic status made many of them unable to afford a radio or television set. As a result, they failed to follow family planning information disseminated via these sources:

*We know that we can also get such information from the radio or television. However, we do not have any of them. We cannot just go to other people’s homes to listen to their radio or watch television just for family planning information*. *It is a disgrace* (FGD 2 – Bariadi).

#### Religious beliefs

Religious beliefs were also found to affect women’s family planning literacy. In this regard, study participants reported that some religions discouraged them to use family planning because doing so prevented eggs fertilization by the sperm thus preventing pregnancy. This, according to them, is against God’s will for people to fill the earth. Family planning thus constitutes interfering with God’s plan:

*Our religion does not allow us to use any form of family planning. Using it is like killing your eggs and your unborn child. That is why you will never hear any Imam or Sheikh in the mosque preaching to his followers about using family planning. That is why many people are not even interested in finding information on it* (FGD 1 – Bukoba).

*Our religious leaders discourage us from using family planning. They tell us that our religion [Roman Catholicism] prohibits the usage of such services [contraceptive]. They want us to continue giving birth because that is what we have been created for…giving birth* (FGD 2 – Musoma).

This situation, as recounted by the women, discourages them and their male partners from seeking any family planning-related information and, as a result, their family planning literacy remains low. In consequence, some women and their partners do not use any kind of recommended modern family planning methods.

### Fertility preference

There were also contrasting views among study participants on the role of fertility preference in their family planning literacy. Some of the study participants indicated that there is a relationship between desire to have large a family and inadequate family planning literacy. Such desire is linked to preference of some family members, particularly male partners, and the community to have many children. FGD participants reported that such a factor influenced women and their male partners to ignoring various family planning messages they receive from different channels:

*It is not that we don’t want to use family planning. The problem is that our spouses want to have many children. They say that their families had only a few children and most of them were females. So, they bear the responsibilities of developing their clans. Therefore, they don’t want to hear anything about family planning* (FGD 2 – Shinyanga rural).

In contrast, other women in the FGDs insisted that many couples now prefer having fewer children as compared to the past. The women attributed these changes to low-income levels, which made using family planning an attractive proposition. They generally believed that having many children could impair their economies and make them fail to take care of their families:

*To be honest, life is very difficult. How are you going to raise all your children during these difficult times? How are you going to provide for them? It’s time now we stop that kind of thinking. Having many children in the family is a burden. We have started thinking about using family planning. We just want to have a small manageable family* (FGD 1 – Bukoba).

### Negative perceptions of and misinformation on family planning

Findings from this study also reveal that there were still many negative perceptions of and misinformation on family planning among many women in rural areas. As a result, women’s efforts to seek accurate family planning information remained severely limited which affected their family planning literacy. A widely held perception among most of the study participants was that many modern family planning methods have negative side-effects harmful to the body:

*We think there is confusion when it comes to using of family planning. It might be true that we have never used any family planning methods before, but what we hear from other people, especially those who have tried to use them, is that these methods have many side-effects on those using them* (FGD 1– Shinyanga rural).

The family planning side-effects they mentioned include continuous bleeding, irregular menses, cancer, loss of sexual desire, swollen stomachs, weight gain or loss, conceiving a disabled child, and becoming infertile. In fact, there was a consensus among study participants that, to a large extent, these perceptions affected their understanding of modern family planning methods hence contributing to their family planning low usage:

*Another side-effect that most people talk about is the danger of women developing cervical cancer. They say that once you start using the pills, your belly will start swelling…that is when you know that you have problems. As a result, many people discourage their use* (FGD 2 – Musoma).

*At our school, we have a special program for disabled children. Now if you try to trace the source of many children’s disabilities, you will find that they are a result of their mothers’ use of syringes [contraceptives] when they were pregnant. So, the syringes affected the fetus in the womb and that is why the children were born disabled* (FGD 2 – Bariadi).

These misconceptions instill so much fear in women such that their utilization of the various family planning methods remains low. Comparatively, many of these misconceptions were more apparent in Musoma district than in the other three districts under review.

### Use of unproven family planning methods

Consistent use of unproven methods of family planning, especially local concoctions and magic (that have not been scientifically proven) was also reported to affect the family planning literacy of some women of childbearing age in some of the communities. This problem was reported more in Musoma district than in three other districts. Some women in this district reported preferring using concoctions from traditional healers than the recommended modern family planning methods. Such concoctions served as an alternative free of side-effects believed to be common among many modern family planning methods:

*Someone may tell you that she took a pill and then her belly started to swell…others may say they have bled the whole month after using some modern family planning methods, or they were very thin but after using the pills they suddenly started gaining weight. So, these things are real. You may ask why you should expose yourself to all these problems when there are other safer options from traditional healers* (FGD 1 – Musoma).

*We normally go to traditional healers for their concoctions…we would rather use the concoctions than those medicines from a health center. At least the concoctions won’t make us infertile like the medicines from the health center* (FGD 2 – Musoma).

*Why should I kill myself with the pills from the hospital? We already know that they are unsafe. If you are using herbals your belly will not swell, you will not gain weight, and there will be no over bleeding. Your body will just be the same* (FGD 1– Bariadi).

From the discussions of study participants, it emerged that some women also believed in magic as a way of controlling unwanted pregnancies. In this regard, the women reported that if they wanted to prevent pregnancies, they went to traditional healers (normally older women) who then drew a tattoo-like mark on their back and inserted a small stick believed to have power to prevent conceiving. The difference between this method and the modern implant is that the one who inserted the traditional remedy had the power to remove it from the woman before she can get pregnant again. Such a woman would only get pregnant when such a traditional healer removes the stick and not otherwise:

*Unlike modern family planning methods, this method has no side effects. The only problem with it is that when you want to remove the stick, it must be removed by the one who inserted it; not anyone else* (FGD 2 – Musoma).

Although most of study participants admitted to having inadequate family planning literacy, they questioned the use of such methods to prevent unwanted pregnancies. The women argued that it was risky to trust a traditional healer on matters related to family planning. The women also revealed that, sometimes a woman might have such a stick installed but still get pregnant. So, in cases like that, doing so is as good as not using any form of family planning.

Overall, findings from FGDs indicate that the belief in and use of concoctions and other unproven family planning methods have made some women less interested in seeking information on modern family planning methods while resorting to such traditional methods. Nonetheless, as noted earlier, the women’s use of these unproven family planning methods is attributable to negative perceptions and fear of potential side-effects of various modern family planning methods.

### Limited access to reliable sources of family planning information

Inability to access family planning-related information from reliable sources was also mentioned by respondents as one of the factors that affected their family planning literacy. This problem was attributed to the shortage of health facilities and limited number of professional health service providers in some remote rural areas. This is especially true for all women staying far from village centers where health care facilities are available. These women use informal sources of information to meet their various health information needs, including family planning ones, as a result. Some study participants said that they usually consulted their immediate relatives or drug sellers in drugstores for such information:

*Sometimes it is difficult to get information on family planning, especially for us who stay far from the health center. So, if we have not gone to the health center but still need to know something about our health or any family planning issue, we will ask our mothers about it. If they are not around, we will just go to the drugstores and ask the sellers about it.* (FGD1 – Bariadi).

Generally, findings from all FGDs indicate that the absence of health facilities in some of the remote rural areas led many women in these areas to turn to drugstore attendants for health information. However, the problem with these sources, as narrated by some of the participants, is that they do not always provide relevant family planning information.

### Household responsibilities

Study participants also mentioned household responsibilities as one of the factors that affected their family planning literacy. Generally, during the discussions it emerged that their engagement in various household activities robbed them of enough time to engage effectively in seeking information on family planning. Although the women admitted that sometimes such information was disseminated through various mass media sources such as radio and television, they said that their tight schedules made it difficult for them to get information from these sources:

*Living in rural areas presents challenges. As women, we engage in every activity in our households…from taking care of children in the house to working in our family farms. You hardly get time to listen to the radio, let alone go to the clinic for family planning services* (FGD 1– Musoma).

When asked about accessing family planning information through mobile phones (which could help them access such information at their convenience), the women reported being unaware of such services despite having access to such devices. Furthermore, those that said they were aware of such services mentioned that they had either not subscribed to such services or subscribed but ignored the family planning messages sent to their mobile phones. Again, the women cited household chores as an obstacle to accessing such information:

*We have subscribed to that service. But as you know, sometimes we might be too busy to check messages when we hear notifications on our mobile phones, we just ignore them…the day may end without reading the messages* (FGD 2 – Bariadi).

### Men’s involvement in family planning matters

Almost all the study participants acknowledged the importance of their partners’ involvement in family planning particularly in enhancing their family planning literacy. The women recounted that their partners’ involvement in family planning helped them as couples to make decisions together on using the recommended family planning methods. The women also mentioned their male partners likelihood to adhere to various family planning instructions and information given by health service providers if they are involved in family planning matters from the very beginning:

*If your partner is also involved in family planning matters, let’s say, you are both provided with family planning information, it will be easier for him to adhere to the requirements because he is also informed. In fact, you are rest assured that once you forget anything, he will be the one to remind you about it* (FGD 1– Shinyanga rural).

*Yes, they may allow us to go to the clinic for family planning services, but the problem here is that we might not tell them exactly what we have been taught at the clinic. If we go together, they will also have the same opportunity as ours, to listen to health service providers about family planning. This helps us as couples to make decisions together regarding whether to use a certain family planning method or not. Otherwise, if they refuse to attend, they are also discouraging us from attending* (FGD 2 – Musoma).

These narrations imply that women whose male partners are supportive of reproductive health solutions were more likely to have adequate family planning literacy than those with unsupportive partners. Yet, only a few study participants reported receiving such support from their male partners. As it would be expected, these women admitted that they were able to discuss various family planning matters with their male partners:

*They are very supportive. We usually talk about it… they even accompanying us to the clinic.* (FGD 1 – Shinyanga rural).

In contrast, many women involved in the study mentioned poor involvement of their male partners in family planning matters as one of the major factors limiting their family planning literacy. These women revealed that their male partners were not supportive of family planning:

*He kept on yelling at me…* “*So, you want me to carry your handbag and go with you to the clinic? Am I the one who want to use family planning? Didn’t you hear that such things have many side effects? Do you want also to get such problems?*” *That is what he was telling me... At such a point, I didn’t have anything to say. I just sat down and cried. I was so disappointed* (FGD 2 – Bukoba).

The women associated their male partners’ poor support for family planning-related matters with their fear of potential side effects of modern family planning methods, preference to have many children, and low levels of education. Consequently, the males discouraged their partners from using family planning methods. Eventually, the women failed to seek family planning-related information such that when the information is sought, it is done without the knowledge of their male partners.

## Discussion

In this study, a qualitative approach has been used to explore the perspectives of women of childbearing age on factors affecting their family planning literacy. Evidence from numerous studies [30, 38, 39] shows that adequate family planning literacy is one of the most important determinants of family planning uptake among women of childbearing age. This study identified a multitude of factors that affect the family planning literacy of women of childbearing age in the studied communities. The findings reveal a significant effect of socioeconomic inequalities on the study participants’ family planning literacy. This is clear when it comes to the extent of unmet family planning information needs among women with no education and those with poor economic status compared to those with at least a primary level of education and above, and those with a better economic status. In fact, the study shows that though most of women who had no education were unable to follow and comprehend various family planning information, those with poor economic status failed to access information disseminated through the mass media sources such as radio and television. In this study, possession of these media sources has been found to be directly linked to one’s economic status. Evidence from previous studies [6, 40] shows that education and wealth have an association with levels of family planning literacy of women in rural areas. However, this study’s revelation that poor household economic status has influenced some women in the communities under review starting to seek family planning information is an encouraging development. This clearly boosts family planning literacy which is likely to translate into uptake of various recommended family planning methods. Nevertheless, findings from other contexts such as coastal regions of Tanzania show that regardless of economic status or level of education, having many children in a family is considered as a prestige [34]. This, therefore, suggests that context might also be one of the factors that affect the family planning literacy of women of childbearing age.

Consistent with findings from other studies [18, 41], this research has also found that religious beliefs negatively affected the family planning literacy of women of childbearing age. Some women that participated in this study treated the usage of family planning as going against God’s will for people to give birth and replenish the world. The women cited their religious leaders’ discouragement of using of family planning as an obstacle to their family planning information seeking. This, however, might be due to varying interpretations of religious texts concerning family planning by some religious leaders as noted in a prior study [41]. According to the study, while some religious leaders encourage the use of family planning, others do not [41]. This situation limits the family planning literacy of women and puts them in a dilemma as to whether they should use family planning or not. This finding illustrates the influence that religious leaders have on their followers when it comes to matters related to family planning.

Unexpectedly, although the women in this study admitted to being restricted by their religious leaders to use family planning, the study’s findings show that some of them still use unproven methods of family planning such as concoctions and magic. The women attributed their preference for unproven family planning methods to fear of potential side effects of modern methods. As a result, these women fail to initiate any efforts aimed to seek information on modern family planning methods. This, however, is not only against their religious beliefs, but also the consistent use of unproven methods put them at more risk than using the modern ones. This finding is similar to those of a study in Nigeria which also reported women’s use of unproven family planning methods [42]. Nevertheless, in the present study most of study participants disapproved the usage of these methods, citing risks the methods might present to their reproductive health. However, although only a minority of study participants confirmed the use of unproven family planning methods, this finding cannot be ignored since these women live in the same communities with the majority that do not use the method. As such, their beliefs are likely to defuse to other women and negatively influence the communities’ family planning literacy.

Despite various efforts aimed to provide communities and women with family planning-related information [43], this study has found that there are still unmet needs for this important information in most of the communities under review. The findings demonstrate that there are still many misconceptions on family planning that affect women’s understanding of various modern family planning methods. In fact, many women and their partners still associate modern family planning methods with potential side-effects such as cancer, over-bleeding, infertility, irregular menses, having disabled children, and swollen stomachs. Such misconceptions discourage women from using different forms of modern family planning. These findings, however, are not peculiar to this study considering that numerous previous studies have also reported the same [3, 18, 30, 44].

The shortage of primary health care facilities and limited skilled personnel in some remote rural areas was also reported to affect the family planning literacy of women of childbearing age in this study. Because of such shortage, women in these areas reported relying on their immediate family members and drugstore attendants available in their villages as their sources of family planning information. These sources, however, might not have any formal training on health matters for them to provide relevant health information to the women as reported in various past studies [45–47]. Reliance on these sources, therefore, puts women at risk of receiving irrelevant family planning information which may contribute to their low uptake of various family planning methods. Unlike the findings in the present study, studies done elsewhere have reported women’s reliance on health workers, television, and radio for family planning information [6, 20].

As noted in this study, there was a common consensus among the women that their engagement in various household activities also limits their family planning literacy. Such engagements denied them sufficient time to engage effectively in seeking family planning information. Although the women admitted that they could also get such information from the mass media sources such as radio and television, they argued that being engaged in various household activities deters their access to these sources of information. It appears that some women in this study had limited exposure to mass media sources. This, as a result, limits their exposure to various family planning messages disseminated through these media.

It is also important to note that, although there is increasing evidence that mobile phones have the potential of improving family planning literacy through family planning text messages [48, 49], a greater proportion of this study’s participants reported not being aware of such an opportunity. The findings demonstrate further that those who were aware of the opportunity and had subscribed to family planning text message services tended to ignore the messages sent to them through their mobile phones. This course of action amounts to a missed opportunity by the women since this service could help them receive family planning information at their convenience and, as such, allow them to improve their family planning literacy. Therefore, there is a need to strengthen campaigns geared towards sensitizing women in rural areas to use their mobile phones to access various health information, including that of family planning.

While male’s involvement in family planning is essential in increasing the support their female partners need in the overall uptake of various recommended family planning methods [50], this study’s findings illustrate poor male involvement in family planning matters generally. Specifically, study participants cited poor involvement of their male partners in family planning as one of the major stumbling blocks to their family planning literacy and overall utilization of various recommended modern family planning methods. Various other studies [38, 50, 51] have also reported similar findings. Like in the aforementioned studies, this study’s participants attributed this problem to their partners’ limited awareness of matters related to family planning, fear of side effects of modern family planning methods, and preference for having many children. As a result, many men are reported to prevent their female partners from engaging in family planning matters.

Furthermore, the study findings show that male partners’ preference of many children in their families made their female partners ignore family planning messages that are disseminated through different channels altogether. Consequently, women’s family planning literacy remains low. The study findings echo those from previous studies [1, 34, 41] that have documented the role of men as the sole decision-makers in matters related to family planning. These findings also signal the influence men have on their female partners’ overall acceptance of various modern family planning methods. This problem, however, can be explained by the fact that for quite a long time, many family planning programs had been focusing on women, thus, excluding men [52, 53].

## Conclusion

Adequate family planning literacy among women of childbearing age is essential in their general uptake of various modern family planning methods. The findings of this study demonstrate a multitude of factors that affect women’s family planning literacy. These factors limit the women’s overall utilization of modern family planning methods. As such, addressing them is imperative in enhancing the effective utilization of modern family planning methods by women. Raising the general literacy level of women in rural areas by reinforcing campaigns geared towards increase their education level is pivotal for them to comprehend various family planning messages from different sources. Efforts to integrate family planning education and information with religious teachings should also be strengthened to eliminate social dilemmas preventing women and their male partners from adopting family planning. Furthermore, improving women’s and their partners’ access to family planning information and encouraging continuous and constant exposure can reduce misconceptions couples have on family planning, thus significantly changing their attitudes and increasing acceptance of various recommended family planning methods. This study supports several initiatives aimed to provide men with family planning education and information. Future research can explore the perspectives of men on their overall uptake of various family planning methods.

## Data Availability

The authors wished to include the guide as suggested, however, the guide contains some more questions which are yet to be used for different manuscripts. As those manuscripts are underwriting, we deem it inappropriate to upload the guide. However, the datasets used and/or analyzed during the current study are available from the corresponding author on reasonable request.
